# Ironic or Overcompensation Effects of Motor Behaviour: An Examination of a Tennis Serving Task Under Pressure

**DOI:** 10.3390/bs9020021

**Published:** 2019-02-20

**Authors:** Recep Gorgulu

**Affiliations:** Elite Performance in Sport Research Group, Faculty of Sport Sciences, Bursa Uludag University, Bursa 16120, Turkey; gorgulurecep@gmail.com

**Keywords:** performance, anxiety, instruction, ironic error, overcompensation, mental control

## Abstract

With specific regard to the hypothesized effects of anxiety on performance in motor behaviour, the rival predictions emanating from the Wegner’s “ironic processes theory” and the “implicit overcompensation hypothesis” are largely indiscriminate. Specifically, Wegner’s theory predicts that self-instructions not to perform in a certain manner would lead to the very behaviour the individual seeks to avoid under pressure. On the other hand, the implicit overcompensation hypothesis predicts that avoidant instructions would produce the opposite outcome to that intended by the performer under pressure. The present novel study directly compared these predictions using a tennis serving task under manipulated instructions. The sample comprised 32 (20 men, 12 women; *M_age_* = 20.81, *SD* = 2.20) experienced tennis players who performed a tennis serving task. Participants’ levels of cognitive anxiety, somatic anxiety and self-confidence were measured by using Mental Readiness Form-3. A 2 (anxiety: low, high) × 3 (serving zone: target zone, non-target ironic error zone, non-target non-ironic error zone) repeated measures of ANOVA revealed a significant anxiety × serving zone interaction *F*(2, 62) = 32.27, *p* < 0.001 which provides specific support for the Wegner’s ironic processes of mental control theory rather than implicit overcompensation hypothesis. More specifically, Bonferroni-corrected follow-up paired samples *t*-tests revealed that when instructed not to serve in a specific direction, anxious performers did so a significantly greater number of times (*t*_31_ = −5.15, *p* < 0.001). The present research demonstrates that ironic performance errors are a meaningful and robust potential concern for performers who are required to perform under pressure.

## 1. Introduction

Imagine a tennis player preparing herself for the critical serve (match point for the opponent), and instructing herself to “serve to her (opponents’) backhand, but not hit wide of the tramlines (out)”. Then imagine her anguish five seconds later, as her serve falls wide of the tramlines, causing her to lose the match. A less severe error, such as making a weak serve to the centre of the court or serving box, would at least have given her a chance of playing a rally, winning the point and staying in the match, but instead she made the mistake she least wanted to make, the so-called *ironic error* [1]. Incidents of ironic errors are thought to be frequent—many people will be able to recall a time when they did the precise thing that they wanted to avoid—especially in pressure-filled environments [2]. For example, an individual may use an inappropriate word that they were trying to suppress during a job interview. Similarly, an Olympic pentathlete revealed that “in some circumstances (e.g., anxiety, pressure), my intention is not to do the best but to avoid making a bad shot. That is when I make a bad shot. When I think about avoiding the error, I make the error” [3] (p. 252). Furthermore, under certain conditions, the inability to managing pressure in sport is strongly associated with sporting performance by increasing level of anxiety, decreasing thought control and as a result of decreased performance expectancies [4]. There have been various theories to explain the manner in which excessive pressure can act to break down human performance. With specific regard to the hypothesized effects of anxiety on performance, the theory of ironic processes of mental control [2] and the De la Pena et al’s [5] implicit overcompensation hypothesis are largely indiscriminate. That is, both theories propose contradictory explanations that anxiety impairs efficient functioning of processing thoughts that leads to the performer to focus on thoughts that will be detrimental to their performance [6,7]. 

Wegner’s [2] theory of the ironic process of mental control explains the “tendency to feel, act, and think in ways that are opposite to the intended direction of emotion, behaviour, and cognition” [1] (p.202). More specifically, Wegner’s [2] theory proposes that when performers’ brain seeks to make the body perform in an intentional way, the brain requires dual processed in order to work efficiently and overcome the performers’ desired state of mind. These two processes are defined by Wegner [2] as the operating process and the monitoring process. First, the operating process aims to create the desired state of mind. For example, this process consciously searches for and directs the person toward areas of sensation and memory of the brain that relate to the intentional state of mind. As a result, this mentally demanding process increases the likelihood of the desired state which will be achieved by the conscious operating process [2,8].

Second, the subconscious monitoring process searches for signals of failure, more specifically these are undesired actions or thoughts. If this subconscious monitoring process identifies any such failures then it immediately reactivates the conscious operating process, which aims to bring about the regulation by filling the mind with mental contents that are more relevant to the desired state. The mental capacity required by both processes under normal conditions fluctuates and adapts, depending on such factors as the task required [2,9]. In daily life, this dual process works automatically and operates as of a feedback loop that allows people effective mental control [2]. However, under certain conditions such as competition, exam or giving a speech in front of people, there is a limited cognitive space for the effortful conscious operating process to work effectively [2]. The theory holds that under conditions of reduced cognitive capacity, the monitoring process may have a greater allocation of cognitive resources focused upon it, and thus will outweigh the operating process [10]. It is in these situations that the ironic monitor comes to the forefront, and duly causes increased sensitivity to actions that are the ironic opposite of those intended: counter-intentional errors [2,10]. Therefore, the subconscious monitoring process becomes more prevalent when pressure is on for one’s and mental control begins to work against itself by attending to those undesired actions [2]. 

Previous research has shown that focusing on instructions to guide thoughts and actions, especially avoidant instructions, can lead to performance outcomes that ironically are contradictory to our intentions (i.e., engaging in the behaviour one was trying to avoid). For example, Wegner and colleagues [8] provided support in a golf-putting task when instructed “don’t overshoot the glow spot”. Results revealed that under high cognitive load, participants put the ball past the spot significantly more often, than those under a low cognitive load condition [8]. However, using the number of retention tasks to induce cognitive load is not related to golf or any other sport; therefore, that type of laboratory task leads to a lack of ecological validity.

Although the previous research [7,11,12,13] provided considerable support for Wegner’s [2] theory, De la Pena and colleagues [5] revealed conflicting results. For example, in their investigation golf players who were instructed not to putt short of the target overcompensated when cognitively loaded (e.g., visual, cognitive, auditory, or self-presentation) and putted significantly farther than under conditions of no cognitive load. However, in this investigation, using the visual, auditory type of methods to induce cognitive load lacks an ecological validity similar to Wegner and colleague’s examination [8]. Malhotra and colleagues [14] provided support for the implicit overcompensation hypothesis in highly automatized skills in driving when drivers were given avoidant instructions (e.g., stay away from the centreline); the results revealed over compensatory behaviour and therefore participants drove further away from the centreline. 

Moreover, time pressures inherent in motor tasks likely present an additional load e.g., Ref [15] which could increase the likelihood of ironic errors under pressurized situations. For instance, police and military marksmen may have less than one second to identify an armed on-rusher as friend or foe and to react accordingly. The consequences of an ironic error in this situation could be grave. Indeed, annual reports of police performance in the United States show that while police officers perform relatively well on low-pressure shooting tests (with hit percentages above 90%), they perform substantially worse when engaged in firing in the line of duty under high-pressure (with hit percentages around or even below 50%; e.g., Ref [16] for a review see Ref [17]). The opening example of a tennis player hitting her return wide of the tramlines is another example of an ironic error during a time-constrained motor task.

However, research to date is limited that has demonstrated an overcompensatory effect in response to avoidant instructions [5,18] especially under ecologically valid experiments and pressure (e.g., mental load). For example, De la Pena and colleagues [5] stated that in their investigations, methods of inducing mental load may have failed to sufficiently tax participants’ cognitive resources. Woodman and colleagues [7] acknowledged that future investigations of ironic processes of mental control theory [2] should continue to ensure that participants’ cognitive resources are significantly taxed in an ecologically valid manner. Therefore, to increase scientific rigour and ecological validity, the aim of the current study was to examine the effects of avoidant instructions and to compare these predictions using a tennis serving task. When serving in tennis, we often see unforced double fault errors or unintended serves (e.g., hitting the undesired side of the court or hitting the net) during the game; however, we do not know are these errors occur due to simply a part of general performance deterioration that can be caused by an overriding implicit counter massage or ironically and specifically due to undesired state of mind. The main research question of the present research is: are these pressure-induced performance failures associated with specifically and precisely ironic effects or activating an overriding implicit counter message? 

## 2. Materials and Methods

### 2.1. Participants

The sample comprised 32 (20 men; 2 left-handed, 18 right-handed, 12 women; all right-handed; *M_age_* = 20.81, *SD* = 2.20) undergraduate student tennis players (*M_years of experience_* = 8.37, *SD* = 2.32) who voluntarily participated in this study. Participants were approached via university coaches and all participants reported being free from illness and injury at the time of the data collection. An informed consent form was obtained from all participants individually. An institutional ethical approval was granted by the local institution (Bursa Uludağ University, Faculty of Medicine Ethics Committee, Ethics number: 2018-4-36) for the current study. 

The GPower 3.1 [19] power calculation software indicated that by adopting an alpha of 0.05 and a sample size of 32 the experiment was powered at 0.80 to detect significant differences between conditions for effect sizes exceeding *f* = 0.20 (i.e., small-to-medium size effects), by repeated measures analysis of variance [20]. While there are limited previous data upon which to base these calculations, Woodman et al.’s [7] test of ironic effects, adopting a similar design, revealed large within-subject effects (ηp2’s = 0.25). Accordingly, if similar effects were to emerge here, this study was more than adequately powered to detect them. 

### 2.2. Measures

#### 2.2.1. Anxiety

Mental Readiness Form-3 [21] used to measure cognitive, somatic anxiety and self-confidence. The MRF-3, which comprises three single-item factors, requires participants to express how they feel right now by placing a mark on three separate 10 cm visual-analogue scales. From left to right the scales are anchored: not worried–worried (cognitive anxiety); tense–not tense (somatic anxiety), and confident–not confident (self-confidence). Thus, high scores represent high cognitive (state) anxiety and low scores represent low cognitive (state) anxiety at the current time of the experiment. The MRF-3 has been widely used by recent research [7,11,15] to evaluate anxiety in competitive settings. This measure was shown to have moderate to strong correlations (r^2^ = 0.58–0.63) with the Competitive State Anxiety Inventory–2 (CSAI-2) [22].

#### 2.2.2. Performance

**Figure 1:**
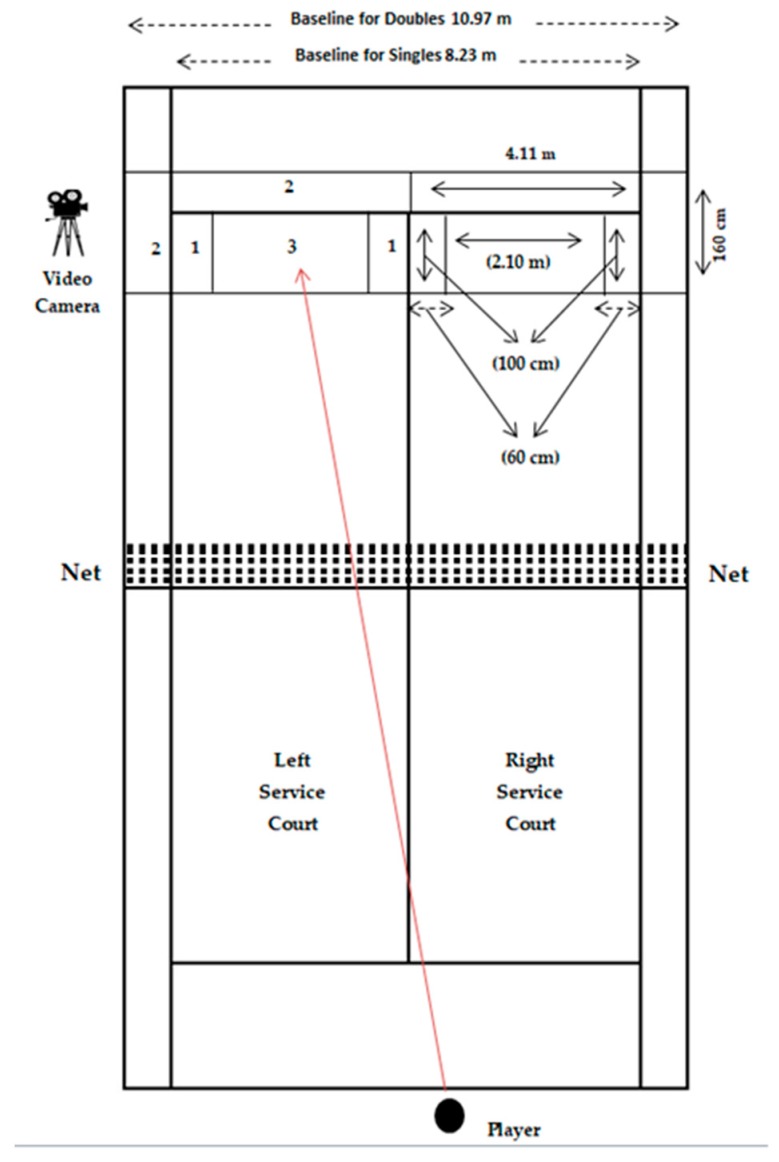
Experimental set up on the tennis court. (Number of the zones are indicated as 1 = target zone, 2 = non-target ironic error zone and 3 = non-target non-error zone.).

Performance was measured by using an ecologically highly valid tennis serving task under different conditions. First, the tennis serving box was conceptualized as a target zone, non-target non-ironic error zone and non-target ironic error zone (Figure 1). The zones were measured with a tapeline and were marked out by the service lines, and each serve was recorded with a high-speed video camera (Phantom Miro V2511, Vision Research, USA). To measure the linear kinematics such as the position, direction and velocity of the tennis ball, the camera was used with a frame rate of 500 Hz and an exposure time of 1/20.000 s. The position of the camera is illustrated in Figure 1. Consequently, participants scored +1 point for serving into the target zone (see Figure 1, zone number 1) and scored minus −1 points for serving out (ironic error zone) or hitting the net (see Figure 1, zone number 2) and 0 points for serving into the non- target non-ironic error zone (see Figure 1, zone number 3). Based on this scoring system, the highest scorer would get 20 points and the minimum point would be 0 in this task. All participants performed the serving task individually (the order of the presentation of the right and left side of the service court was counterbalanced; therefore, each player served an equal number of serves to each side of the tennis court). Participants were allowed to use their own racquets as they wished, and US Open brand new tennis balls were used for each participant starting from the first until the end of the third trial; therefore, each participant opened a new tennis ball box to use over the course of their experiment. Serve accuracy was estimated by the speed of the ball and balls that landed outside the court after the second bounce were measured to control the speed of the ball and to make sure each serve was performed accurately with the maximum serving speed of the performer. 

### 2.3. Procedure

Participants arrived at the indoor tennis court individually. The experimenter informed each participant about the procedure and described the scoring system before asking to complete the questionnaire pack. Next, participants completed an informed consent and demographic information sheet (e.g., age, sex, year of experience in sport). Before the experiment, participants were allowed to warm up as they usually do before the training session or an actual competition (e.g., tournament). 

Participants then were given an instructional set as “Please try to serve into the target zone to get 1 point for each ball; however, please be careful not to serve into the net or out as you will score −1 point for each ball and finally, for any ball you hit within the serving box rather the target zone, you will get 0 points”. The instructional set to which the participant had been assigned was repeated immediately prior to commencement. This was repeated again halfway through, to ensure that the participant had not forgotten and had remained alert. The experimental procedure consisted of three trials followed by a ten-minute break between each trial. First, participants completed a familiarization trial comprising 10 serves with the scoring system and serving zones as a warm-up. This allowed them to become more accustomed to the nature of the task and allowed the researchers to verify that participants understood the instructional set before the main experiment under low-and high-pressurized instructional trials. Upon completion of the first trial (warm-up) participants responded to the MRF-3 and then performed 20 serves under the low-anxiety conditions in the second trial. The experimenter followed the same procedure in the last trial with one exception that before completing the MRF-3, participants were informed that they were about to enter the competition by participating in this research and the highest scoring participant would receive a brand new tennis racquet (worth approx. $150) as a present from the researcher. The aim was to manipulate anxiety using multiple ecologically valid performance stressors (i.e., competitive environment, financial incentive, and social evaluation), [7,11,22,23]. Then, participants performed 20 serves under the high-anxiety condition. Eventually, participants were informed that their scores during the performance and all serves were observed as beyond controversy (by using the video recording system for each ball land to the court) while entering one of the three distinct areas. On completion of the experiment, the participants were thanked for their participation and were asked not to disclose any information regarding the experiment.

### 2.4. Ethics

All procedures performed in studies involving human participants were in accordance with the ethical standards of the institutional and/or national research committee and with the 1964 Helsinki declaration and its later amendments or comparable ethical standards. An institutional ethical approval was granted by the local institution of Bursa Uludağ University, Faculty of Medicine Ethics Committee with the Ethics number of 2018-4-36, for the current study.

## 3. Results

### 3.1. Anxiety Manipulation

Self-report MRF-3 data confirmed the effectiveness of the successful anxiety manipulation check. Specifically, results obtained from the MRF-3 demonstrate an expected increase in participants’ cognitive anxiety (*t*31 = −6.61, *p* < 0.001) and somatic anxiety (*t*31 = −9.34, *p* < 0.001) from low- to high-anxiety condition, and participants’ self-confidence decreased from low to high-anxiety conditions (*t*31 = 9.62, *p* < 0.001). The results for the anxiety manipulation check are summarized in Table 1.

### 3.2. Performance

A 2 (anxiety: low, high) × 3 (serving zone: target, non-target ironic error, non-target non ironic error) repeated measures of Analysis of Variance (ANOVA) was performed. This yielded a significant main effect for condition (anxiety; low-high) *F*(1, 31) = 43.28, *p* > 0.001, a significant main effect for serve, *F*(2, 62) = 49.45, *p* < 0.001, and a significant anxiety × zone interaction, *F*(2, 62) = 32.27, *p* < 0.001. The sphericity assumption was violated, therefore a Greenhouse-Geisser corrention factor to the degrees of freedom was applied in the current study. Accordingly, Bonferoni-corrected follow-up paired samples *t*-tests revealed that when instructed not to serve in a specific direction on the tennis court, anxious performers did so a significantly greater number of times (*t*_31_ = −5.15, *p* < 0.001). Importantly, there was no difference in non- target non-ironic error (all *ts* < 1, *ps* > 0.5), which provides specific support for Wegner’s theory [2,10] in a performance setting (Table 2). 

## 4. Discussion

The primary goal of the present study was to perform a direct test of the movement predictions for the implicit overcompensation hypothesis and ironic processes theory of pressure-induced performance errors. As hypothesized, under the high-anxiety condition, compared to the low-anxiety condition, participants’ ironic error hits were significantly farther from the target zone and significantly farther into the ironic error zone. In other words, when anxious, participants performed ironically [7] which provides support for the Wegner’s [2] theory rather than an implicit overcompensation hypothesis. More specifically, results revealed that when instructed not hit into the certain part of the serving box, tennis players hit significantly more balls into the incorrect zone under pressure. 

This result contrasts the predictions made by the theory of the implicit overcompensation hypothesis. According to De la Pena et al. [5], negative or unintentional instructions may lead to overcompensation effect of the performers. For example, in their investigation, De la Pena and colleagues [5] found that the instruction “not to undershoot” creates an implicit message that it is better to do in an opposite way that ends up in overshooting the target. This clearly would lead performers to an implicit overcompensation of movement rather than the ironic effect of doing something unintentionally. However, this is not an issue in the present study; the results demonstrated that when anxious, participants served significantly more serves into the avoided zone (e.g., zone number 2). More specifically, despite manipulating the task instruction and creating the pressurized situation for tennis players, their target serves and non-target non-ironic error serves did not change across anxiety conditions. Previous research provided support for Wegner’s theory [2] and found a similar effect for the non-target ironic error; however, this is the first study that did not find any difference in target serves from low- to high-anxiety conditions. The findings of the present research provide better support for Wegner’s theory as the only change was specifically for the non-target ironic error zone. Consequently, the results of the present study clearly explain that given instruction not to serve in a certain place may ironically lead performers to serve exactly into the non-target ironic error zone under pressure. Malhotra and colleagues [14] found opposite results in a driving motor control task that avoidant instructions did not lead participants to ironic behaviours. In the same study, researchers reported that the overcompensatory effects of behaviour were present only when driving without cognitive load (e.g., tone counting). In other words, avoidant instructions led drivers to overcompensate when driving with no cognitive load. However, an important difference between the theories is that Wegner’s [2] theory predicts that performance when cognitively loaded (e.g., anxious) will break down in a precisely ironic manner (e.g., hitting the golf ball in the water—the one spot to avoid). As a result, given an avoidant instruction with no cognitive load may not lead participants to ironic behaviour and equally may not provide clear comparative results for these theories. 

Indeed, when exploring Wegner’s [2] ironic processes of mental control theory in motor control and sports settings, it is essential to note that this is not very different in nature to basic cognitive models of attention and interference, e.g., Ref [24,25], that can be used in other domains such as education, business and military services. Such models suggest that in evaluative settings, performance levels under pressure will be reduced [26] in other domains too. Specifically, these models maintain that more errors are made as cognitive resources are diverted away from the task by cognitively distracting responses such as worry, emotionality and task generated interference [27]. More specifically, the states of mind people feel before and during task engagement allow them to perform tasks of varying natures and difficulties such as those encountered in the sporting environment [28]. Therefore, it posits that the unwanted performance errors may be generated by having to respond to factors such as worry and anxiety, causing an overload on cognitive resources, to the point at which capacity to focus on performance suffers, and thus more errors are made. These assumptions need to be explored by future research in different contexts. 

From the applied perspective, results emanating from the current study suggest that in order to reduce an athlete’s susceptibility to counter-intentional errors, task instructions given to them should remain simple, positive and action based. This should be particularly apparent under pressure situations, as negative instructions would leave the performer susceptible to counter-intentional errors caused by a monitoring process. It should also be particularly apparent when educating novice performers that non-experts may not possess the psychological skills to deal with adversity [1]. Therefore, if negative instructions are given to especially novice performers, they may again be particularly susceptible to counter-intentional errors under pressure due to the limited space for the operating process. 

Notwithstanding, there remain some limitations and unanswered questions to be tackled by future research. First, although the large within-subject effects (ηp2’s = 0.25) was revealed in the method section of the current study, this study included a relatively small sample size; therefore, using larger sample sizes would be worthwhile to increase statistical power. Second, while a tennis serving task was chosen due to its suitability for evaluating ironic effects in a popular sport, future investigation should employ more complex motor tasks involving attentional demands of the movement. The current research lacks attentional measures such as probe reaction time that requires participants to perform a choice reaction task involving the coordination of multiple joints, as occurs in sport. Lastly, the incidence of counter-intentional errors in relation to gender differences was not explored in the current study due to its relative lack of female participants and to date, there is no study that has explored this. This is particularly apparent based on findings that the self-report of anxiety symptoms and overall anxiety sensitivity of female performers is significantly higher than that of male performers, e.g., Ref [29]. If this is the case, then the ironic processes theory suggests that female performers may be more susceptible to counter-intentional errors than males. This is because the additional anxiety females are under would place a greater demand on cognitive resources and thus allow a greater possibility to increase non-target ironic errors. Thus, an interesting route to pursue would be possible gender differences in the incidence of counter-intentional errors in future.

## 5. Conclusions

Regardless of the explanation used to account for the occurrence of counter-intentional errors, Wegner’s [2] ironic processes of mental control theory suggests that performers are at greater risk of succumbing to counter-intentional errors when under increased cognitive demand. The present research demonstrates that ironic performance errors are a meaningful and robust potential concern for performers who are required to perform under pressure. Regardless, coaches and practitioners would do well to be particularly careful with the specific words that they use as part of their instructions when helping performers to ensure that they do not contribute to the likelihood of mental control backfiring [5] when it matters most to the performer (e.g., in competition). In this premise, results emanating from the current study would suggest to all performers that they make sure to employ appropriate psychological techniques to keep stress and anxiety levels under check before a competition or important event.

## Figures and Tables

**Table 1:** Descriptive statistics of the Mental Readiness Form-3.

	Condition		
Measure	Low-Anxiety	High-Anxiety	
	Mean (*SD*)	Mean (*SD*)	*t*(31)
Cognitive Anxiety	5.00 (*1.54*)	7.87 (*1.97*)	−6.61 ***
Somatic Anxiety	4.81 (*1.92*)	8.21 (*1.71*)	−9.34 ***
Self-confidence	7.43 (*1.74*)	4.25 (*1.52*)	9.62 ***

Note: *** = *p* < 0.001.

**Table 2:** Mean number of serves (SD) in the target, ironic and non-target non-ironic error zones, under the low-anxiety and high-anxiety conditions.

	Condition		
Measure	Low-Anxiety	High-Anxiety	
	Mean (*SD*)	Mean (*SD*)	*t*(31)
Target Zone (1)	10.34 (*2.41*)	9.90 (*2.65*)	1.91
Non-target ironic error (2)	4.46 (*1.79*)	5.37 (*1.91*)	−5.15 ***
Non-target non-ironic error (3)	5.06 (*2.03*)	4.75 (*2.12*)	1.66

Note: *** = *p* < 0.001, Number of zones are indicated in Figure 1 as 1 = target zone, 2 = non-target ironic error zone and 3 = non-target non-error zone.
